# Biomonitoring of Dietary Mycotoxin Exposure and Associated Impact on the Gut Microbiome in Nigerian Infants

**DOI:** 10.1021/acs.est.3c07786

**Published:** 2024-01-22

**Authors:** Kolawole I. Ayeni, David Seki, Petra Pjevac, Bela Hausmann, Magdaléna Krausová, Dominik Braun, Lukas Wisgrill, David Berry, Benedikt Warth, Chibundu N. Ezekiel

**Affiliations:** † Department of Microbiology, Babcock University, Ilishan Remo PMB 4003, Ogun State, Nigeria; ‡ University of Vienna, Faculty of Chemistry, Department of Food Chemistry and Toxicology, Währinger Straße 38, Vienna 1090, Austria; § Joint Microbiome Facility of the Medical University of Vienna and the University of Vienna, Djerassiplatz 1, Vienna 1030, Austria; ∥ Division of Clinical Microbiology, Department of Laboratory Medicine, Medical University of Vienna, Vienna 1090, Austria; ⊥ Department of Microbiology and Ecosystem Science, Centre for Microbiology and Environmental Systems Science, University of Vienna, Djerassiplatz 1, Vienna 1030, Austria; # Division of Neonatology, Pediatric Intensive Care and Neuropediatrics, Comprehensive Center for Pediatrics, Department of Pediatrics and Adolescent Medicine, Medical University of Vienna, Vienna 1090, Austria; ¶ Exposome Austria, Research Infrastructure and National EIRENE Node, Vienna 1090, Austria; ∇ University of Natural Resources and Life Sciences Vienna (BOKU), Department of Agrobiotechnology (IFA-Tulln), Institute for Bioanalytics and Agro-Metabolomics, Konrad-LorenzStr. 20, Tulln 3430, Austria

**Keywords:** early life exposure, food safety, gastrointestinal microbiome, contaminants, chemical exposome, sub-Saharan Africa

## Abstract

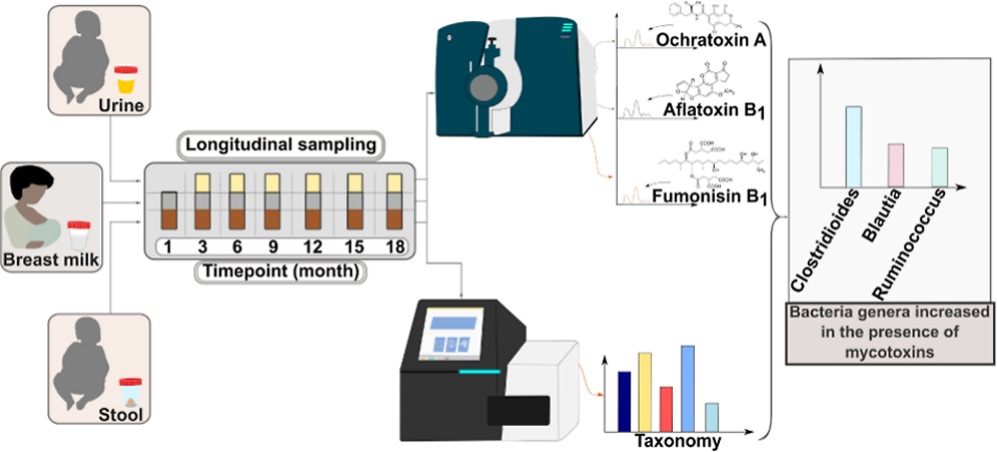

Mycotoxins are toxic chemicals that adversely affect human health. Here, we assessed the influence of mycotoxin exposure on the longitudinal development of early life intestinal microbiota of Nigerian neonates and infants (NIs). Human biomonitoring assays based on liquid chromatography tandem mass spectrometry were applied to quantify mycotoxins in breast milk (*n* = 68) consumed by the NIs, their stool (*n* = 82), and urine samples (*n* = 15), which were collected longitudinally from month 1–18 postdelivery. Microbial community composition was characterized by 16S rRNA gene amplicon sequencing of stool samples and was correlated to mycotoxin exposure patterns. Fumonisin B_1_ (FB_1_), FB_2_, and alternariol monomethyl ether (AME) were frequently quantified in stool samples between months 6 and 18. Aflatoxin M_1_ (AFM_1_), AME, and citrinin were quantified in breast milk samples at low concentrations. AFM_1_, FB_1_, and ochratoxin A were quantified in urine samples at relatively high concentrations. *Klebsiella* and *Escherichia*/*Shigella* were dominant in very early life stool samples (month 1), whereas *Bifidobacterium* was dominant between months 3 and 6. The total mycotoxin levels in stool were significantly associated with NIs’ gut microbiome composition (PERMANOVA, *p* < 0.05). However, no significant correlation was observed between specific microbiota and the detection of certain mycotoxins. Albeit a small cohort, this study demonstrates that mycotoxins may influence early life gut microbiome composition.

## Abstract 2

Mycotoxins were detected in biological samples of Nigerian neonates and infants, and concentrations in stool were associated with altered gut microbiome composition.

## 1 Introduction

The concept of the “developmental origins of health and disease” (DOHaD) postulates that early life exposure to toxic chemicals can negatively influence health outcomes later in life.^1,2^ Mycotoxins are toxicants of high relevance to this concept, owing to their relatively high abundance in food crops, prevalence of ingestion, and high toxicity.^3,4^ Ingestion of mycotoxin-contaminated foods can lead to negative health outcomes such as stunting and cancers.^4^ Mycotoxins of toxicological importance include aflatoxins (AF), fumonisins (FBs), ochratoxin A (OTA), citrinin (CIT), deoxynivalenol (DON), and zearalenone (ZEN).^4^ In addition, *Alternaria* mycotoxins [e.g., alternariol and its monomethyl ether (AME)] are attracting research attention due to technological advancement in liquid chromatography with tandem mass spectrometry (LC–MS/MS) methods that enable quantification of a multitude of mycotoxins in a single analytical run.^5,6^

In the gastrointestinal tract, mycotoxins can exhibit toxicity^7^ by compromising the gut barrier integrity^8^ and modifying the gut microbiota (GM).^9^ As the microbiota can also modify mycotoxins, there is a complex bidirectional relationship between the mycotoxins and the GM.^10^ In humans, the GM is involved in several crucial roles such as digestion of food,^11^ immune system maturation,^12^ and prevention of pathogen colonization.^13^ However, under dysbiotic conditions, specific members of the GM have been associated with severe malnutrition^14^ and gastrointestinal diseases.^15,16^ Mycotoxins might drive dysbiotic development of neonatal GM, yet the underlying mechanisms remain poorly understood. Many published studies on the interaction between the GM and the mycotoxins are based on animal models or human cell lines. For instance, it was observed that OTA decreased the GM diversity in rats^17^ and aflatoxin B_1_ (AFB_1_) reportedly restricted cell growth in a Caco-2 colon cell model.^18^ Conversely, in vitro incubation with *Alternaria* mycotoxins resulted in modifications of the mycotoxins.^9^ There is a lack of information about the interactions between the mycotoxins and the early life GM in vivo.

Several studies have described human GM development in African,^19,20^ Asian,^21^ and Western cohorts.^22−26^ Neonates and infants (NIs) in sub-Saharan African (SSA) countries such as Nigeria are frequently exposed to mycotoxins^4^ that may potentially influence early life GM.^27^ Still, to our knowledge, no study has so far assessed the development of early life GM in a mycotoxin-exposed NIs’ population. In addition, there is a paucity of data on mycotoxin exposure patterns of NIs anchored on data from stool and breast milk over 18-month postdelivery. Therefore, the objectives of this study were to assess mycotoxin exposure patterns in NIs using three biospecimens (breast milk, stool, and urine) and to determine the GM composition of NIs in a mycotoxin-exposed population. Subsequently, the potential associations between mycotoxin exposure and GM development were assessed in a longitudinal setting during the first 500 days of life.

## 2 Materials and Methods

### 2.1 Study Area and Population

In total, 14 healthy mother–infant pairs recruited from southwest Nigeria (Ilishan-Remo and Ojota) participated in this study. These locations were selected based on (a) previous studies that revealed high occurrence and concentrations of mycotoxins in complementary foods^28,29^ and human breast milk,^30^ and (b) lack of longitudinal microbiome data for NIs. The most common complementary foods consumed in the study locations are cereal-based (e.g., maize, sorghum, *ogi*), nut-based (groundnut), and mixed cereal- and nut-based foods (*tombran*, granola).^30,31^

### 2.2 Study Design and Ethical Approval

The present study is a pilot longitudinal biomonitoring study that was conducted between January 2019 and May 2021. Pregnant mothers were recruited from Babcock University Teaching Hospital or local households. Participation was voluntary after proper education on the purpose of the study was given. Each mother provided written informed consent prior to the study inclusion. Inclusion criteria were healthy mothers above the age of 18 who gave birth to a healthy infant and were willing and able to donate biospecimens. Authorization to conduct the study was obtained from the National Health Research Ethics Committee of Nigeria through the Babcock University Health Research and Ethics Committee (BUHREC). The approval numbers are BUHREC585/18 and BUHREC421/21R.

### 2.3 Sampling

Samples of food (breast milk and complementary/solid foods) consumed by each NI, as well as stool and urine excreted, were collected at 3 months sampling intervals up to 18 months postdelivery. Data on mycotoxins in complementary/solid foods fed to infants was published elsewhere.^31^ NIs’ stool samples were collected by mothers from diapers. First morning urine samples from NIs were collected by mothers. Breast milk samples were collected by mothers as well, matching the time-point of stool and urine sample collection. Breast milk (10 mL) and stool (5–10 g) samples were collected in sterile 25-mL sample bottles. Each stool sample was aliquoted into two batches: batch A for mycotoxin analysis and batch B for 16S rRNA gene amplicon-based microbiota analysis. Batches A and B of stool samples together with breast milk and urine samples were transported in a cold chamber at 4 °C prior to storage at −20 °C until analysis.

Not all participants provided samples at every time point. Detailed distribution of all samples collected is shown in Table S1. Overall, the number of samples collected from participants in this pilot longitudinal study were breast milk (68), stool (82), and urine (15) (Table S1). In total, 165 biological samples were collected and analyzed.

### 2.4 Chemicals and Reagents

Acetonitrile (ACN), methanol (MeOH), and water were purchased from Honeywell (Seelze, Germany). Ammonium acetate, formic acid, anhydrous magnesium sulfate, sodium chloride, and formic acid were purchased from Sigma-Aldrich (Vienna, Austria). Details of all reference and internal standards (IS) are reported elsewhere.^32,33^ All solutions and solid reference were stored at −20 °C.

#### 2.4.1 Multimycotoxin Analysis of Breast Milk Samples

Mycotoxins in the breast milk samples were analyzed using the method described by Braun et al.^32^ Briefly, samples were thawed and homogenized, and 1 mL of breast milk sample was extracted with 1 mL of acidified ACN (1% formic acid). After homogenization (3 min), anhydrous magnesium sulfate (0.4 g) and sodium chloride (0.1 g) were separately added, and the sample was homogenized by vortexing (3 min). The supernatant was transferred into a microreaction tube after a centrifugation step (4750 × *g*, 10 min, 4 °C). The extract was chilled at −20 °C for 24 h following a second centrifugation step (14,000 × *g*, 2 min, 4 °C). The supernatant was diluted in a H_2_O preloaded reservoir to 5% ACN and subsequently loaded to an Oasis PRiME HLB solid phase extraction (SPE) column (1 cc, 30 mg, Waters, Milford, MA). This column was equilibrated with 1 mL of ACN and conditioned with 1 mL of H_2_O/ACN (95/5, v/v) prior to sample loading. Following sample loading, the column was washed twice (5% ACN, 500 μL) and mycotoxins were eluted using pure ACN. This extract was evaporated to dryness using a vacuum concentrator (Labconco, Missouri, USA) and samples were reconstituted in 81 μL MeOH/ACN (50:50, v/v) and 9 μL of the IS mixture.

#### 2.4.2 Multimycotoxin Analysis of Urine Samples

Urine samples were subjected to multimycotoxin analysis following the stable-isotope dilution assay-based LC–MS/MS method developed by Šarkanj et al.^34^ and expanded by Braun et al.^35^ Briefly, each urine sample (1 mL) was thawed and centrifuged for 3 min at 5600 × *g*. Afterward, the samples were treated with β-glucuronidase from *E. coli* Type IX-A (Sigma-Aldrich, G7396-2MU) prior to SPE cleanup on Oasis PRiMEHLB SPE columns (Waters, Milford, MA, USA). Extracts were then evaporated to dryness in a vacuum concentrator (Labconco, Missouri, USA) reconstituted with 490 μL of dilution solvent (10% acetonitrile, 0.1% acetic acid) and fortified with 10 μL of IS mixture.

#### 2.4.3 Multimycotoxin Analysis of Stool Samples

The method of Krausová et al.^33^ was applied to quantify mycotoxins in stool samples. Briefly, samples were thoroughly homogenized with a sterile spatula, and an approximately 40 mg aliquot was transferred into an Eppendorf tube (2 mL) followed by a drying step (24 h) in a vacuum concentrator (Labconco, Missouri, USA). Thereafter, water was added (40 μL per 20 mg), followed by vortexing. Extraction solvent (ACN/MeOH/HAc, 49.5/49.5/1, v/v/v) was added (160 μL per 20 mg), followed by thorough vortexing and ultrasonication in an ice-bath for 15 min. The samples were stored overnight at −20 °C to precipitate proteins. Thereafter, samples were centrifuged at 18,000 × *g*, for 10 min at 4 °C, after which the supernatants were transferred to new reaction tubes. The supernatants were diluted (1:10) with water containing 0.1% HAc and 10 μL of IS mixture was added. Then, the diluted samples were filtered through polytetrafluoroethylene filters. The resulting overall dilution factor of this procedure was 1:100.

#### 2.4.4 LC–MS/MS Analysis

After sample extraction, mycotoxins in breast milk, urine and stool were measured using a Sciex QTrap 6500^+^ LC–MS/MS system (Foster City, CA) equipped with a Turbo-V ESI source coupled to an Agilent 1290 series UHPLC system (Waldbronn, Germany) of the Exposome Austria research infrastructure using validated methods.^32,33,35^

### 2.5 Culture-Independent Analysis of Stool Samples

#### 2.5.1 Stool DNA Extraction

The total genomic DNA from stool samples was extracted using the QIAamp Fast DNA Stool Mini Kit following the manufacturers’ protocol. Negative control extractions (extraction kit reagents) were included to allow for assessment of contamination.^36^

#### 2.5.2 16S rRNA Gene Amplification

The V4 region of the 16S rRNA gene of most bacteria and archaea was amplified using the primer pair 515F (GTG YCA GCM GCC GCG GTA A) and 806R (GGA CTA CNV GGG TWT CTA AT),^37,38^ in a two-step PCR protocol as described by Pjevac et al.^36^

#### 2.5.3 16S rRNA Gene Amplicon Sequencing

Amplicon sequencing was carried out at the Joint Microbiome Facility (JMF) of the Medical University of Vienna and University of Vienna under JMF Project ID JMF-2105-01. Indexed sequencing libraries were prepared with the Illumina TruSeq Nano Kit and sequenced in paired-end mode (600 cycles; V3 chemistry) on an Illumina MiSeq following the manufacturers’ instructions. The workflow systematically included four negative controls (PCR blanks, i.e., PCR-grade water as a template) for each 90 samples sequenced. Also, a ZYMObiomics mock community was sequenced and analyzed at the sequencing facility as part of established quality control routine.^36^

#### 2.5.4 Amplicon Sequence Data Analysis

Amplicon pools were extracted from the raw sequencing data by using the FASTQ workflow in BaseSpace (Illumina) with default parameters. Demultiplexing was performed with the python package demultiplex, allowing one mismatch for barcodes and two mismatches for head sequence and primers. Barcodes, linkers, and primers were trimmed off by using BBDuk. Amplicon Sequence Variants (ASVs) were inferred using the DADA2 with R version 3.6.1^39^ applying the recommended workflow.^40^ FASTQ reads were trimmed at 220/230 nt with allowed expected errors of two-fourths. ASV sequences were subsequently merged and classified using SINA version 1.6.0^41^ and the SILVA database SSU ref NR 99 release 138 using default parameters.^42^ Processed data were further analyzed using the following R packages: ampvis2,^43^ dplyr,^44^ ggplot2,^45^ phyloseq,^46^ tidyr,^47^ vegan,^48^ pheatmap,^49^ and MaAsLin2.^50^ Raw sequence reads generated in this study are available under BioProject accession number PRJNA1013123.

### 2.6 Statistical Analysis

Species richness (observed ASVs and Chao1) and diversity (Shannon) metrics were compared based on the rarefied ASV subset by nonparametric Wilcoxon test, using ampvis2 in R software version 4.3.1.^51^ Differences in NIs’ stool microbiota by age were determined with Bray–Curtis’ distance. For quantitative mycotoxin analysis, raw data were integrated and quantified using SciexOS (v2.1) software. Data were further analyzed using Microsoft Excel 2016, Origin Pro 2023 (v 9.85.212), and R software (version 4.3.0). A Shapiro–Wilk test was performed to determine normality. Since data were not normally distributed, mycotoxin concentrations in breast milk, stool, and urine were log-transformed, including the addition of a pseudocount: *y* = log 10 (1 + mycotoxin concentration) to create a normal distribution for comparison purpose. Sample concentrations above the limit of detection (LOD), but below the limit of quantitation (LOQ), were set to LOQ/2. Concentrations of mycotoxins in the three matrices were represented using boxplots in Origin Pro 2023 and R studio using the ggplot2 package.^45^ PERMANOVA was used to test for association between mycotoxins and GM composition and visualized using redundancy analysis (RDA) in R. In addition, MaAsLin2^50^ was used for identification of taxa significantly correlated with the presence of mycotoxins.

## 3 Results and Discussion

### 3.1 Characteristics of the Study Participants

Seven male and seven female NIs participated in this study. At birth, the mean ± SD weight of the NIs was 3.2 ± 0.6 kg. There was no significant difference (*p* > 0.05) between the average weight of the male (3.4 ± 0.6 kg) and female (3.1 ± 0.6 kg) NIs. Nine (64%) neonates were delivered via Caesarean section while five (36%) were vaginally delivered. Ten neonates (69%) were exclusively breastfed for the first six months of life, whereas four (31%) were nonexclusively breastfed because they consumed complementary foods in addition to breast milk (Table S2).

### 3.2 Mycotoxins in Breast Milk

**Figure 1:**
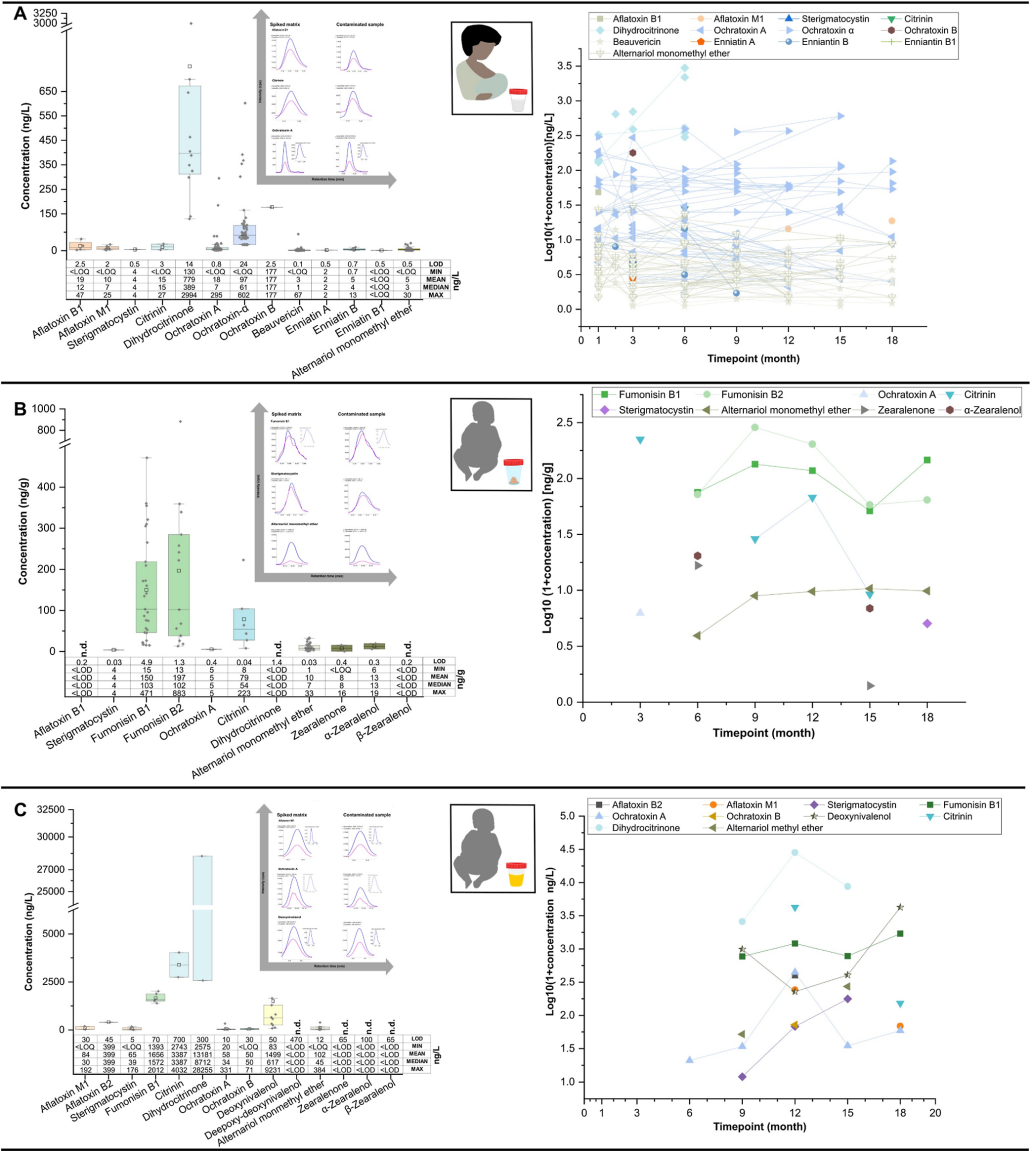
Occurrence and dynamic distribution of quantified mycotoxins in (A) breast milk (*n* = 68), (B) stool (*n* = 82), and (C) urine (*n* = 15) over 18-month postdelivery. Overall prevalence of mycotoxins in the stool coincided with increased cofeeding with complementary/solid foods. MRM-chromatograms of selected mycotoxins in naturally contaminated samples and spiked matrixes are represented for each matrix.

In total, 34 mycotoxins were analyzed, of which 13 were detected in the breast milk samples. This includes AFB_1_, AFM_1_, sterigmatocystin (STC), OTA, ochratoxin α (OT-α), ochratoxin B, CIT, dihydrocitrinone (DHC), beauvericin (BEA), enniatin A (Enn A), enniatin B (Enn B), enniatin B_1_ (Enn B_1_), and alternariol monomethyl ether (AME) (Figure 1a & Table S3). Each breast milk sample contained at least four mycotoxins (Figure S1). The spectra of mycotoxins in breast milk in the present study were similar to a previous study that quantified mycotoxins in breast milk (AFM_1_, AME, BEA, DHC, Enn B, Enn B_1_, OTA, and STC).^30^ Despite applying the same method, AFB_1_, a group 1 carcinogen^52^ was not detected in that study or any other Nigerian breast milk until now. AFB_1_ was detected in 6% of the breast milk at median concentration of 12 ng/L (mean: 19 ng/L; range: < LOQ −47 ng/L). A slightly higher detection rate of 9% (7/78) (range: 56–291 ng/L) was reported for AFB_1_ in breast milk from Ecuadorian mothers.^53^ The Ecuadorian study applied HPLC-FD, which was nine times less sensitive (LOD: 23 ng/L) than our method (LOD: 2.5 ng/L). AFM_1_, a possible carcinogen, was detected in 12% of the breast milk at mean concentration of 10 ng/L (median: 7 ng/L), which was lower than the value of 35 ng/L previously reported in a study from Nigeria,^54^ but higher than the value of 2 ng/L reported in another study from Nigeria.^30^ A Brazilian study reported AFM_1_ in 2% (2/100) of breast milk samples at concentrations of 0.3 and 0.8 ng/L.^55^ STC, a precursor metabolite of AFB_1_ was detected in only one sample at a concentration of 4 ng/L. The nephrotoxin, CIT was detected in 3% of breast milk samples (median: 15 ng/mL; mean: 15 ng/L; range: < LOQ −27 ng/L), but its metabolite, DHC (mean: 779 ng/L; range: 130–2994 ng/L), was detected in 16% of the samples, at median concentration 27 times higher than the value of 14 ng/L previously reported in Nigeria.^30^ Since DHC is suggestive of CIT exposure,^56^ indications are that some of the mothers were likely exposed to high CIT levels.

The median OTA concentration of 7 ng/L (detection rate: 87%; mean: 18 ng/L; range: < LOQ −295 ng/L) in the breast milk samples was about four times higher than the value of 2 ng/L previously reported in Nigerian^30^ and Austrian mothers.^35^ Conversely, median OTA concentration was about seven times lower than the value of 48.3 ng/L (LOD: 18 ng/L) reported in breast milk from Bolivia.^57^ Other OTA metabolites in the breast milk were: OTB, detected in only one sample (177 ng/L), and OT-α, which was detected in 74% of the samples at median concentration of 61 ng/L (mean: 97 ng/L; < LOQ −602 ng/L). Ochratoxin-α is the major metabolite of OTA,^58^ and as such its relatively high concentration in the breast milk is not surprising. The detection rate (91%) and median concentration (3 ng/L) of AME in breast milk were similar to the levels (detection rate: 96%; median: 1.8 ng/L) previously reported in Nigerian^30^ and Austrian mothers (detection rate: 90%; median: 3.6 ng/L).^35^ The median BEA concentration of 1 ng/L (detection rate: 87%; mean: 3 ng/L; range: < LOQ −67 ng/L) in breast milk in the present study was similar to the median concentration of 1.2 ng/L reported in Austria.^35^ In addition to differences in analytical instruments and sensitivities, the differences between the values reported in the present study compared to other studies could be due to seasonal variations, geographical locations, and feeding practices.

### 3.3 Mycotoxins in Stool

To our knowledge, no study has reported multiple mycotoxins in NIs’ stool over 18 months postdelivery. Stool biomarkers are important to human biomonitoring studies because they provide broader insight into human exposure to poorly absorbed mycotoxins such as fumonisins,^33^ and can help track biotransformation products, especially in high-risk mycotoxin regions such as Nigeria. Furthermore, stool sampling is noninvasive, and samples are easy to collect.^33^ Our laboratory previously published data on mycotoxins in stool from the same cohort at month 12.^33^ Thus, only longitudinal patterns of mycotoxins in stool are discussed in detail herein.

**Figure 2:**
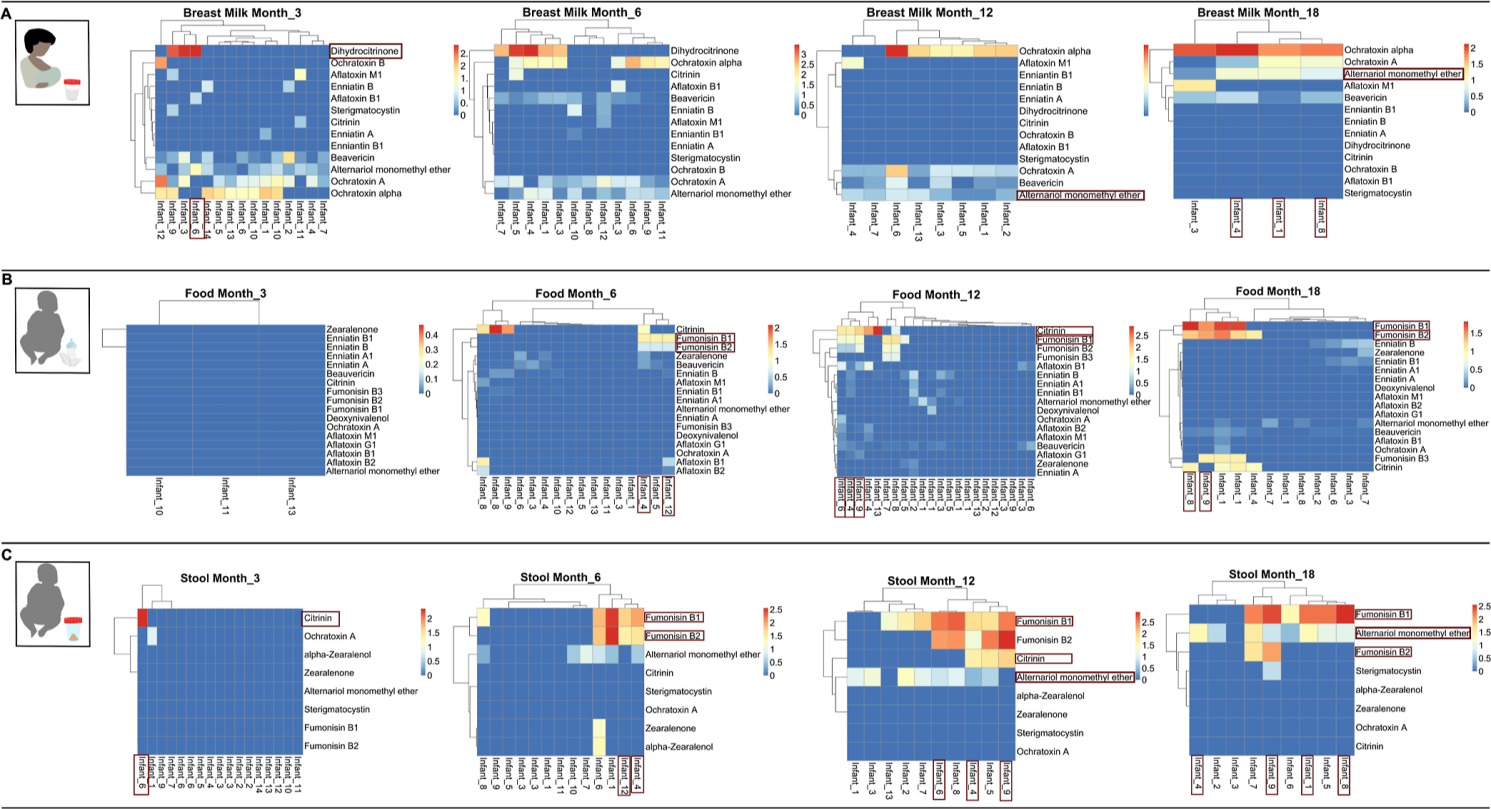
Mycotoxin exposure pattern in (A) breast milk, (B) complementary food (data from Ayeni et al.^31^), and (C) stool displayed for samples collected at selected time points (months 3, 6, 12 and 18). Certain mycotoxins were detected in all matrices at specific time points for a few infants. For instance, fumonisin B_1_ was detected at high levels in food consumed by infant 4 and infant 12 at month 6, which was reflected by high prevalence of fumonisin B_1_ in stool of the same infants. At month 12, a high detection rate was recorded for citrinin in foods consumed by infants 4 and 9 that was reflected by high prevalence of citrinin in stool of the same infants. At month 18, high prevalence of fumonisin B_1_ was detected in food and stool of infant 8.

OTA and CIT were each detected in one sample at month 3 but, the overall prevalence of mycotoxins in the stool coincided with increased cofeeding with complementary foods (Figures 1b and 2). FB_1_, B_2_ and AME were quantified in the stool between month 6 and 18, suggesting continuous exposure during this critical developmental period (Figures 1b and 2). This observation provides strong evidence that complementary foods were the major contributors to mycotoxin exposure in the study cohort. A previous study from the same region reported a higher spectra and concentration of mycotoxins in urine of nonexclusively breastfed children, compared to exclusively breastfed children.^30^ Thus, our findings further underscore breastfeeding as a safe and viable option to reduce mycotoxin exposure during early life in the study region and potentially other high risk mycotoxin exposure regions. Other mycotoxins detected in the stool were STC, ZEN, and α-ZEL, which are to our knowledge reported for the first time in NIs’ stool.

Despite high aflatoxin exposure via consumption of traditionally processed complementary foods,^31^ aflatoxins were not detected in any of the stool samples. A Chinese study, using a more sensitive detection method, reported the presence of AFB_1_ (0.02 μg/kg) in one out of three analyzed stool samples.^59^ We hypothesize that the NIs’ GM may have biodegraded the AFs leaving only trace concentrations or biotransformation products below the LOD for AFs (0.1–2.2 ng/g) of the analytical method applied in the present study. In addition, metabolism of AFB_1_ in the liver and subsequent rapid absorption into the blood,^60,61^ and excretion through the urine^62^ may have contributed to the nondetection of AFs in the stool samples.

### 3.4 Mycotoxins in Urine

A limited number of urine samples were analyzed in the present study mainly due to the difficulty in collection of urine from the NIs. Despite the limited sample size, urine biomarkers are crucial to human biomonitoring studies, since they provide information about recent exposure (24 h) to some mycotoxins.^63^ As such, the presented urine data provide additional valuable insight into the level of mycotoxin exposure among the NIs. Overall, 30 mycotoxins were analyzed in urine samples, and 10 thereof were detected, including AFM_1_, AFB_2_, FB_1_, DON, OTA, OTB, CIT, DHC, STC, and AME (Table S4 and Figure 1c).

AFM_1_ was detected in three urine samples at median concentration of 30 ng/L (detection rate: 20%; mean: 84 ng/L), which was higher than the value of 24 ng/L and about seven times higher than median value of 4.4 pg/mL reported in urine of infants and young children (IYC) from Nigeria and Bangladesh, respectively.^64,65^ The median FB_1_ concentration of 1572 ng/L (detection rate: 40%; mean: 1656 ng/L; range: 1393–2012 ng/L) detected in the urine was about five times higher than the 301 ng/L value previously reported in nonexclusively breastfed Nigerian IYC.^30^ CIT was quantified in 13% of urine at median concentration of 3387 ng/L (mean: 3387 ng/L; range: 2743–4032 ng/L), which was about 31 times higher than the median value of 0.11 ng/mL reported in urine from Bangladeshi IYC.^66^ DHC was quantified in urine samples at median concentration of 8712 ng/L (detection rate: 20%; mean: 13,181 ng/L; range: 2575–28,255 ng/L), which was about three times higher than the parent molecule CIT, and 126 times higher than the median of 69 ng/L previously reported in nonexclusively breastfed Nigerian IYC (detection rate: 74%; mean: 202 ng/L; range: 5–1377 ng/L).^30^ Furthermore, the DHC urine level was about 19 times higher than the median value of 0.47 ng/mL reported in Bangladeshi IYC urine.^66^

DON and OTA were detected in 67 and 73% of urine at respective median concentrations of 617 and 34 ng/L. The median DON concentration was lower than the median value of 1 ng/mL reported in IYC urine from Bangladesh,^64^ while median OTA was about five times higher than the median value of 7 ng/L previously reported in nonexclusively breastfed infants in Nigeria.^30^ The high detection rate of OTA in the urine samples in the present study contradicts a recent report from Nigeria, wherein AFQ_1_ and ZEN were the most frequently detected mycotoxins in urine of exclusively and nonexclusively breastfed Nigerian infants, respectively.^30^ In fact, in the present study, neither ZEN nor AFQ_1_ were detected in any urine sample. This observation may be due to the limited number of urine samples analyzed herein. Other mycotoxins detected in the NIs’ urine were STC and AME, which are both reported for the first time in urine of the Nigerian NIs.

### 3.5 Mycotoxins Exposure Dynamics in All Matrices

Heatmaps were created in RStudio to incorporate mycotoxins quantified in all matrices across different time points (Figures 2 and S4). Mycotoxin data in complementary food was obtained from Ayeni et al.^31^ As can be expected, certain mycotoxins that were detected in the NIs’ food (breast milk or complementary food) were also found in stool samples. For example, at month 9, high concentrations of FB_1_ were detected in complementary foods consumed by infants’ 4 and 7, which was reflected by a high concentration of FB_1_ in stool of the same infants (Figure S4). At month 12, high concentration of CIT was detected in complementary foods consumed by infants’ 4 and 9, while high CIT occurrence was recorded in stool of the same infants (Figures 2 and S4). High prevalence of FB_1_ was observed in complementary foods consumed by infant 8 at month 15 and infant 8 at month 18, that was reflected in high FB_1_ detection in stool of the same infants (Figures 2 & S4). AME was detected in breast milk consumed by infants’ 1, 4, and 8 at month 18 that was reflected by frequent detection of AME in stool of the same infants (Figures 2 and S4). These observations showed that dietary exposure patterns of CIT, AME, and FBs can be readily tracked from food to biofluids.

### 3.6 GM Diversity and Community Structure

In view of the potential chronic health effects of dietary chemical exposures such as mycotoxins during early life and the emerging exposome paradigm to improve environmental and public health,^67^ this study further sought to investigate whether mycotoxins in stool influence the GM in the study cohort.

**Figure 3:**
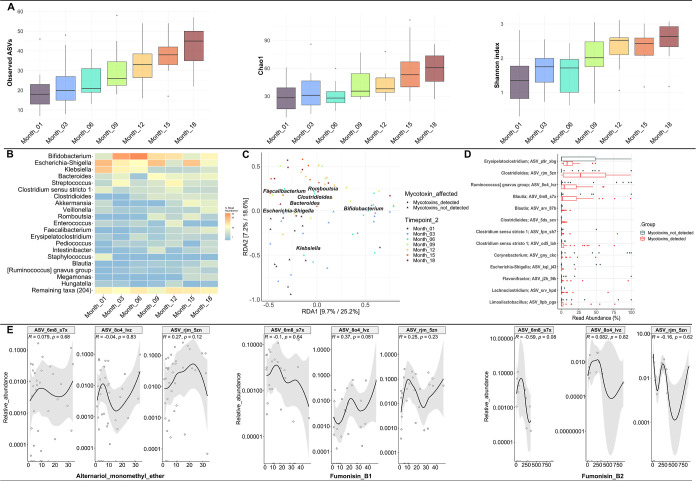
(A) Alpha diversity indices for the bacterial community in stool samples across different time points. Observed ASV and Shannon index revealed significant difference (*p* < 0.05, Wilcoxon test) in stool samples between month 1 and month 18 postdelivery and (B) heatmap of top 20 genera in neonate and infant’s stool (C) redundancy analysis revealed an association between some bacteria taxa and the presence of mycotoxins, (D) taxa belonging to the genera *Clostridioides*, *Ruminococcus*, and *Blautia* were detected at higher relative abundance in stool samples containing mycotoxins, and (E) Spearman correlation analysis revealed no significant correlation (*p* > 0.05) between taxa showing highest relative abundance in the presence of mycotoxins and individual mycotoxins.

Following rarefaction to an even depth (*n* = 2351 reads), a total of 660 different ASVs were obtained from the stool samples. A significant increase in alpha diversity (as measured via the Shannon Index) was observed in samples collected at 18 months postdelivery as compared to one month postdelivery (Wilcoxon test, *p* = 0.041 Figure 3a). Trends in primary succession of neonates and infant’s GM are in line with previous studies that reported an increase in alpha diversity over the NIs’ first year postdelivery.^20,21,25^ Furthermore, age postdelivery was significantly associated with GM composition (PERMANOVA, *p* < 0.05). Bray–Curtis distances within month 1 samples were statistically different from samples from month 3 to 18 (*p* < 0.05).

### 3.7 GM Profile in Neonates’ and Infants’ Stool

At the phylum level, stool samples were on average dominated by *Firmicutes* (34%), *Proteobacteria* (31%), *Actinobacteriota* (28%) and *Bacteroidota* (7%) (Figure S2). When clustered by age postdelivery, *Proteobacteria* dominated month 1 samples, *Actinobacteriota* were predominant in months 3 and 6 samples, and *Firmicutes* were dominant between months 12 and 18 (Figure S3). Overall, 224 different bacterial genera were present in the stool samples, with the most dominant genera being *Bifidobacterium*, *Escherichia*/*Shigella*, *Klebsiella*, *Bacteroides*, *Streptococcus*, *Clostridium* sensu stricto 1, *Clostridioides*, *Akkermansia*, *Veillonella*, *Romboutsia*, *Enterococcus*, *Faecalibacterium*, *Erysipelatoclostridium*, *Pediococcus,* and *Intestinibacter* (Figure 3b). While the prevalence of *Bifidobacterium* was like in several other studies,^20,23,68−70^ some findings differentiate our cohort. For instance, levels of potentially pro-inflammatory or pathogenic bacteria including *Escherichia*/*Shigella* and *Klebsiella* were remarkably high and persistent in stool of some NIs at month 1 postdelivery. These bacteria might contribute to an elevated burden of pro-inflammatory priming during early life in our cohort.

### 3.8 Impact of Mycotoxins on GM Composition

To test whether mycotoxins exerted an effect on NIs’ GM, despite the sparse detection of quantifiable individual mycotoxins across samples, each stool sample was categorized as either having detectable mycotoxins or not. At this level, it was observed that the detection of mycotoxins was significantly associated with GM composition (PERMANOVA, *p* = 0.001). Results were further explored using RDA, guided by constraints of the following significantly associated variables: age, individual NI, and presence of mycotoxins (Figure 3c). A slight clustering in GM composition was observed with the presence of mycotoxins, but it remains unclear from the RDA which bacteria contributed most to the observed variation. This observation must be interpreted with caution since age postdelivery was highly colinear with mycotoxin detection. However, in early time point samples (months three to six), the genus *Klebsiella* slightly clustered with mycotoxin detection (Figure 3c). To further determine which taxa might be associated with overall mycotoxin presence, the MaAsLin2 package^50^ was employed to test for differential abundance according to presence or absence of mycotoxins. Several ASVs were significantly associated with the presence of mycotoxins in stool. Notably, *Clostridioides difficile* [ASV_rjm_5zn (p.adj = 0.0015)] had the highest relative abundance of those significantly associated taxa (Figure 3d). However, none of the associated taxa significantly correlated to any individually quantified mycotoxin levels (Figure 3e), which may be attributable to the small sample cohort and sparse detection of mycotoxins in the infant stool. Mycotoxins were previously associated with immunological dysregulation in animal models.^71,72^ Thus, we do not exclude potential interactions between mycotoxins and the GM, which might lead to shifts in both composition and immunomodulatory activity with potentially adverse consequences. Furthermore, while causal links between the presence of mycotoxins and altered GM composition in vivo remain to be elucidated, it appears that taxa of the genus *Clostridioides* are associated with mycotoxin exposure during early life, showing higher relative abundance in NIs’ stool in the presence of mycotoxins.

### 3.9 Limitations of the Study

While no other study has yet investigated multiple mycotoxin exposure biomarkers in different biological specimens and correlated them with microbiome data in a longitudinal design, this study has several limitations. First, the number of study participants was small (*n* = 14), which was caused largely by challenges with cohort recruitment. In addition, not every participant provided samples at all time points. Second, the approach of “mycotoxin detected” versus “mycotoxin non-detected” applied to explore associations between the infant gut microbiome and mycotoxin exposure is not without its limitation since mycotoxins differ in their mode of action. However, the approach to an extent mimics a real-life exposure scenario since humans are typically exposed to multiple mycotoxins, especially through diet. Third, it was not possible to obtain microbiome data from a similar cohort that consumed a diet completely free of mycotoxin contaminants, which could have served as a control for this study cohort. Lastly, only mycotoxins were analyzed in the biospecimens, but the NIs could have been exposed to several other toxicants that may influence the gut microbiome. However, several challenges can limit microbiome research across SSA, thus understanding the influence of xenobiotics such as mycotoxins on early life GM is still in its exploratory stage.^27,73^ Therefore, the data presented herein is an important step toward understanding in vivo chemical exposome–microbiome interactions.

## 4 Implications

This study provides an important, longitudinal data set on mycotoxin exposure patterns of Nigerian NIs based on stool and breast milk samples. Recognizing that it is difficult to eliminate mycotoxins in foods consumed by NIs in the study region, our data reinforce the recommendation that breast milk nutrition is a safe and viable way to reduce mycotoxin exposure. Thus, exclusive breastfeeding at least within the first 6 months of life and continuous breastfeeding following the introduction of complementary foods for up to two years, as recommended by the WHO, is strongly advised. Future studies may consider an exposome-wide scale approach anchored on nontargeted analysis and suspect screening, to unravel other chemicals the NIs were exposed to, and that may influence the GM.^74^ While the NIs’ stools expectedly contained taxa with beneficial, commensal, and pathogenic potentials, it was surprising that month 1 stool samples were dominated by *Klebsiella*. Thus, future studies should investigate the prevalence of *Klebsiella* in early life stool from the Nigerian NIs and consequent long-term health effects. In addition, interactions between species such as *Clostridioides difficile* and selected mycotoxins should be tested in an in vitro model. Furthermore, it would be worthwhile to conduct large-scale population-based chemical exposome–microbiome studies comparing cohorts across different geographical locations.

## Supporting Information Available

Characteristics of study participant, distribution of mycotoxins in breastmilk, stool, and urine, overview of amplicon reads per stool sample, mycotoxins exposure pattern in matrix provided by each neonate and infant, and distribution of phylum in stool (PDF)
(XLSX)
