# Supplementary material for: Biomonitoring of Dietary Mycotoxin Exposure and Associated Impact on the Gut Microbiome in Nigerian Infants
Source: Environ Sci Technol. 2024 Jan 22;58(5):2236–46. doi: 10.1021/acs.est.3c07786 (PMC10851434; doi:10.1021/acs.est.3c07786)
Supplement: Supplementary file 1 — es3c07786_si_001.pdf [file es3c07786_si_001.pdf]

Supporting Information

Biomonitoring of dietary mycotoxin exposure and associated impact on the gut microbiome in Nigerian infants

Kolawole I. Ayeni[1,2], David Seki[3,4], Petra Pjevac[3,5], Bela Hausmann[3,4], Magdaléna Krausová[2], Dominik Braun[2], Lukas Wisgrill[6,7], David Berry[3,5,#,*], Benedikt Warth[2,7,#,*], Chibundu N. Ezekiel[1,8,#,*]

[1]Department of Microbiology, Babcock University, PMB 4003, Ilishan Remo, Ogun State, Nigeria

[2]University of Vienna, Faculty of Chemistry, Department of Food Chemistry and Toxicology, Währinger Straße 38, 1090 Vienna, Austria

[3]Joint Microbiome Facility of the Medical University of Vienna and the University of Vienna, Djerassiplatz 1, 1030 Vienna, Austria

[4]Division of Clinical Microbiology, Department of Laboratory Medicine, Medical University of Vienna, 1090 Vienna, Austria

[5]Department of Microbiology and Ecosystem Science, Centre for Microbiology and Environmental Systems Science, University of Vienna, Djerassiplatz 1, 1030 Vienna, Austria

[6]Division of Neonatology, Pediatric Intensive Care and Neuropediatrics, Comprehensive Center for Pediatrics, Department of Pediatrics and Adolescent Medicine, Medical University of Vienna, 1090 Vienna, Austria.

[7]Exposome Austria, Research Infrastructure and National EIRENE Node, 1090 Vienna, Austria

[8]University of Natural Resources and Life Sciences Vienna (BOKU), Department of Agrobiotechnology (IFA-Tulln), Institute for Bioanalytics and Agro-Metabolomics, Konrad-LorenzStr. 20, 3430, Tulln, Austria.

[#]Shared correspondence and senior authorship:

chaugez@gmail.com, benedikt.warth@univie.ac.at, david.berry@univie.ac.at

[*]Shared corresponding authorship:

chaugez@gmail.com, benedikt.warth@univie.ac.at, david.berry@univie.ac.at

Summary: 22 pages, 7 figures, 6 tables

Contents

Figure S1. Distribution of mycotoxins quantified in breast milk consumed by each neonate and infant, as well as stool and urine excreted by each neonate and infant. Alternariol monomethyl ether was frequently detected in breast milk and stool from most neonates and infants.
Figure S2. Four dominant phyla in stool samples of Nigerian neonates and infants. Values in pie-chart represent percentage occurrence of each phylum.
Figure S3. Heatmap of dominant phyla in stool of neonates and infants.
Figure S4. Mycotoxin exposure pattern in (A) breast milk, (B) complementary food (data from Ayeni et al.[1]), and (C) stool displayed for all samples collected.

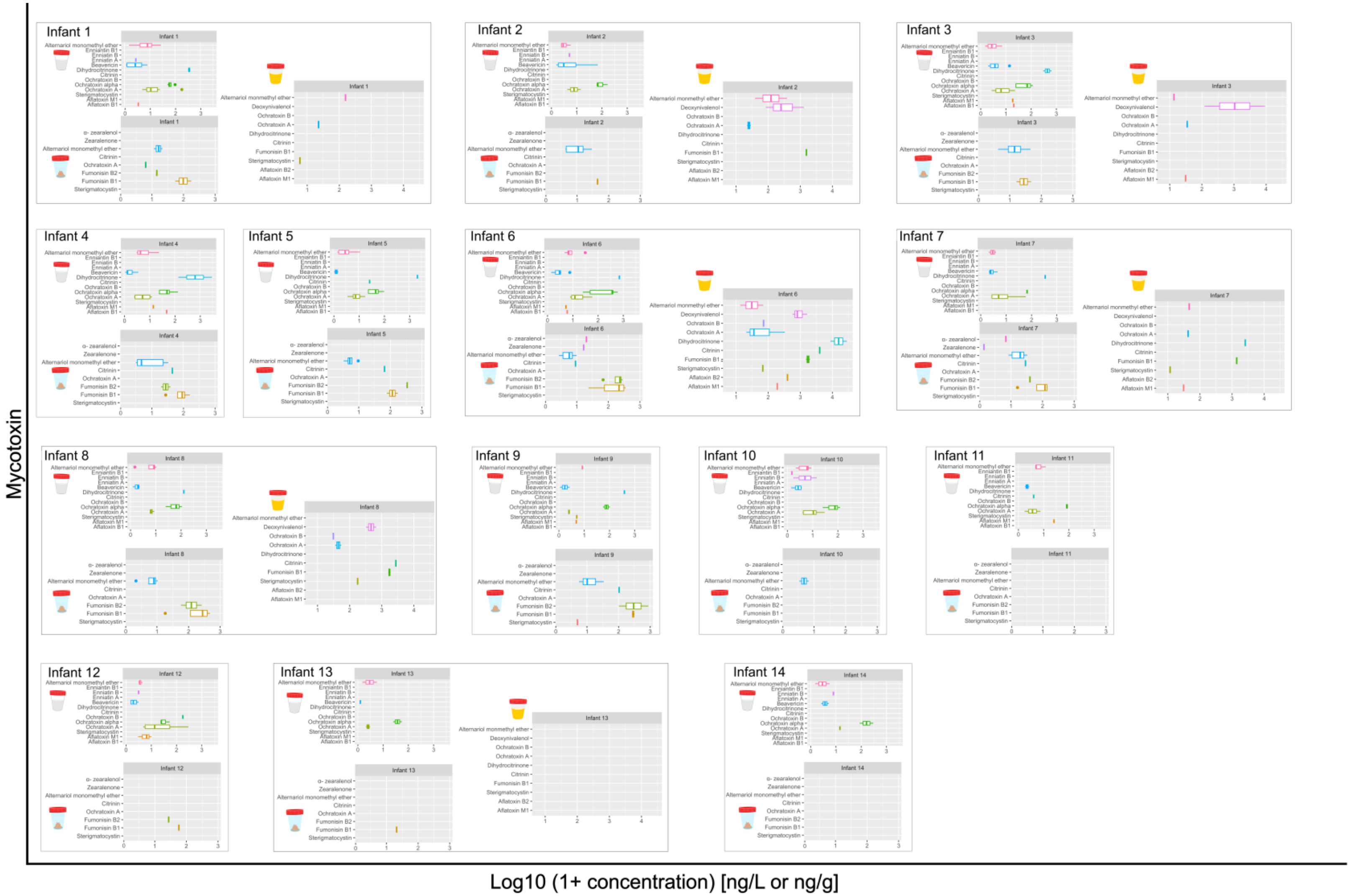

Figure S1. Distribution of mycotoxins quantified in breast milk consumed by each neonate and infant, as well as stool and urine excreted by each neonate and infant. Alternariol monomethyl ether was frequently detected in breast milk and stool from most neonates and infants.

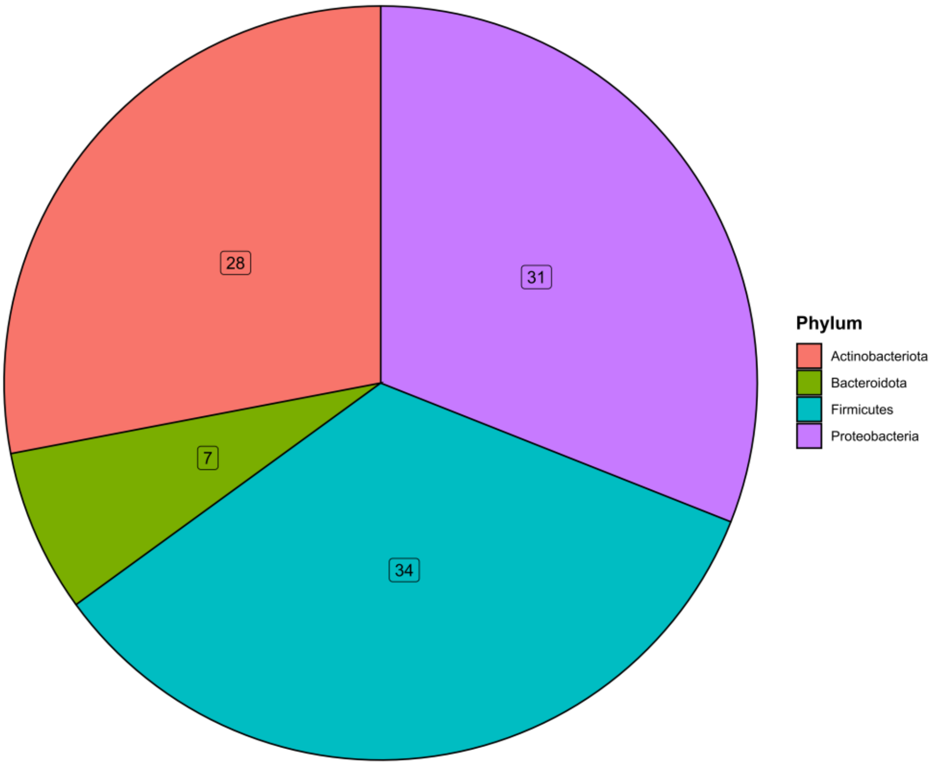

Figure S2. Four dominant phyla in stool samples of Nigerian neonates and infants. Values in pie-chart represent percentage occurrence of each phylum.

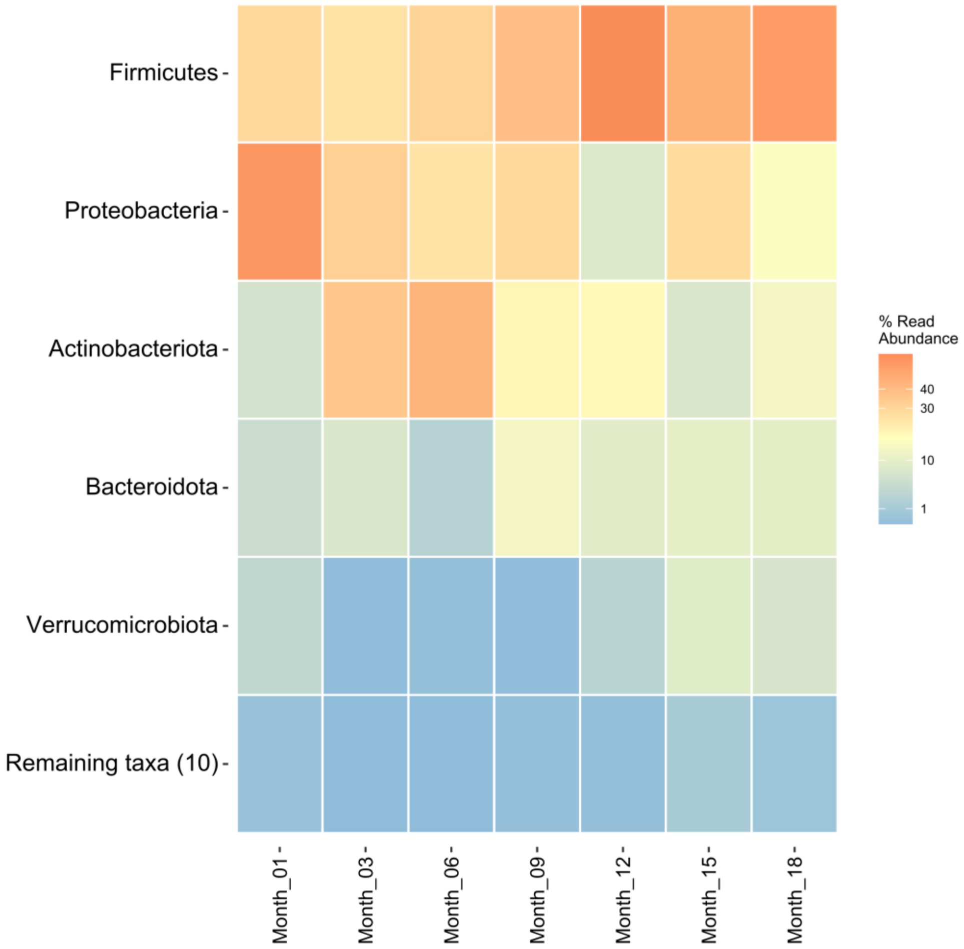

Figure S3. Heatmap of dominant phyla in stool of neonates and infants.

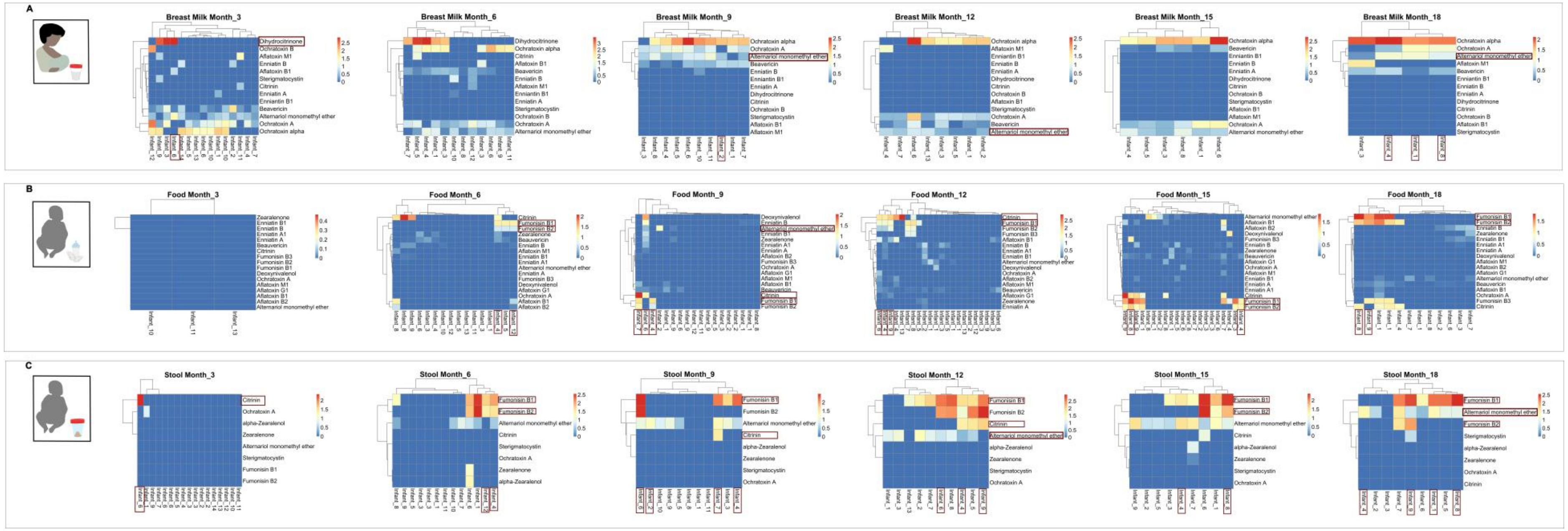

Figure S4. Mycotoxin exposure pattern in (A) breast milk, (B) complementary food (data from Ayeni et al.[1]), and (C) stool displayed for all samples collected.

REFERENCE

(1) Ayeni, K. I.; Sulyok, M.; Krska, R.; Warth, B. Ezekiel, C. N. Mycotoxins in complementary foods consumed by infants and young children within the first 18 months of life. *Food Control* **2023**, *144*, 109328.
